# A computer-assisted metal analyser using flow injection coupled with direct current plasma–optical emission spectroscopy

**DOI:** 10.1155/S1463924690000232

**Published:** 1990

**Authors:** M. C. Brennan, R. A. Simons, G. Svehla, P. B. Stockwell

**Affiliations:** 1 University College Cork Ireland; 2 PS Analytical Ltd Arthur House B4 Chaucer Business Park Watery Lane, Kemsing, Kent Sevenoaks TN15 6QY UK

## Abstract

An automated routine method was developed for the determination of trace metals using a flow injection system into a direct current plasma spectrometer. The characteristic emission in the ‘Spectra- Metrics’ three-electrode jet is channelled through an echelle grating to the detector of the instrument. The emission signal is continuously recorded, and, after digitalization, calculated and recorded by a Touchstone software package and interface card. The software has facilities for recording peaks, generating calibration curves, calculating and printing results, enhancing line sensitivities, monitoring signal response and for filter noise, recalibration, background correction, status monitoring and general diagnostics. Up to 70 elements can be determined both in aqueous and non-aqueous media.


Journal of Automatic Chemistry, Vol. 12, No. 5 (September–October 1990), pp. 183–188

### A computer-assisted metal analyser using flow injection coupled with direct current plasma—optical emission spectroscopy

**M. C. Brennan, R. A. Simons, G. Svehla and P. B. Stockwell**

*University College Cork, Ireland; PS Analytical Ltd, Arthur House, B4 Chaucer Business Park, Watery Lane, Kemsing, Sevenoaks, Kent TN15 6QY, UK; University College Cork, Ireland*

*An automated routine method was developed for the determination of trace metals using a flow injection system into a direct current plasma spectrometer. The characteristic emission in the 'Spectra-Metrics' three-electrode jet is channelled through an echelle grating to the detector of the instrument. The emission signal is continuously recorded, and, after digitalization, calculated and recorded by a Touchstone software package and interface card. The software has facilities for recording peaks, generating calibration curves, calculating and printing results, enhancing line sensitivities, monitoring signal response and for filter noise, recalibration, background correction, status monitoring and general diagnostics. Up to 70 elements can be determined both in aqueous and non-aqueous media.*

### Introduction

Direct current plasma optical emission spectrometry (DCP-OES) offers accurate and precise determination of about 70 elements at trace concentration levels. Its chief advantage in comparison with traditional emission spectrographic methods is that samples can be introduced in solution, thus shortening measurement time and cutting out all the work associated with cleaning and alignment of electrodes, weighing out solid samples and reference materials.

Usually, solutions are sprayed into the plasma in batch operations, either manually, or, with large numbers of samples, with the aid of an autosampler. However, a continuous spraying of liquids into the plasma is quite feasible by using flow injection analysis (FIA) for moving and injecting samples. The first attempt to combine FIA with inductively coupled plasma optical emission spectrometry (ICP-OES) was made as early as 1981 [1]; since then mathematical models have been set up for using the standard addition principle involving FIA-ICP-OES [2, 3]; problems of nebulization efficiency have been examined [4–6]; and even various combinations of liquid chromatography with FIA-ICP-OES systems have been attempted [7–10]. Some reviews on the combination of FIA with atomic spectroscopy are available, which also cover applications for ICP-OES [11–13].

For the routine determination of analytes in the quality control of the production of speciality chemicals the authors have used the combination of DCP-AES with FIA. Results obtained with the determination of boron, copper, molybdenum, tungsten and zinc in non-aqueous solutions have been published elsewhere [14]. Since then the principle has been extended to other analytes, carrier liquids and solvents, and the purpose of the present paper is to give an account on the details of a fully automatic system which has been in continuous use for more than 2 years.

The combination of FIA with DCP-OES means that a constant nebulization is maintained over a longer period of time. The introduction of the sample as a plug into the carrier stream causes a transient signal (peak) in the response, which soon decreases to the background level caused by the carrier liquid [15]. This signal compares well with the continuous nebulization responses which are obtained by nebulizing large volumes of samples over a longer period of time. In a typical application, signals extending to 93% of the continuous signal have been produced, with a relative standard deviation of 1·6% over 20 injections (figure 1).

### Experimental

### *The spectrometer*

The instrumentation used for this study consists of a Beckman Spectrospan 4 direct current plasma – optical emission spectrophotometer. Figure 2 shows a schematic diagram of the DC plasma source that is well suited for excitation of emission spectra for a wide variety of elements. This plasma jet consists of three electrodes arranged in an inverted 'Y' configuration. A graphite anode is located in each arm of the 'Y' and a tungsten cathode at the inverted base. Argon flows from the two anode blocks towards the cathode. The plasma jet is formed by bringing the cathode momentarily into contact with the anodes. Ionization of the argon occurs, and a current of about 15 amps (A) develops which generates additional ions which sustain the current indefinitely. The plasma source can accept liquid samples, either aqueous or non-aqueous, with ease. The sample is aspirated into the area between the two arms of the 'Y', where it is atomized, excited, viewed and measured. The plasma temperature is around 5500° K [16]. This temperature (higher than the maximum of 2600° K used in flame atomic emission and absorption spectrometry) is capable of removing most matrix interferences and chemical effects and improves the sensitivity, thus making the DCP source an extremely versatile tool for a wider range of samples and solvents. The higher thermal energy offers a 10-fold increase in linear calibration range.

0142–0453/90 $3.00 © 1990 Taylor & Francis Ltd.

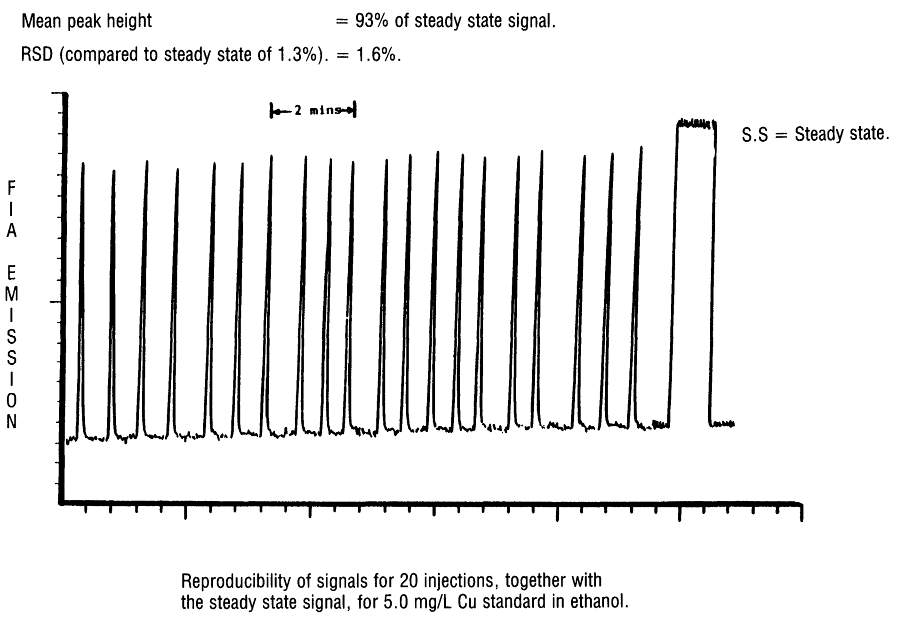

*Figure 1. Reproducibility of signals obtained with 20 injections of 5 μg/cm$^3$ Cu standard in ethanol. Mean peak height = 93% of continuous nebulization. RSD (compared to continuous of 1·3%) = 1·6%.*

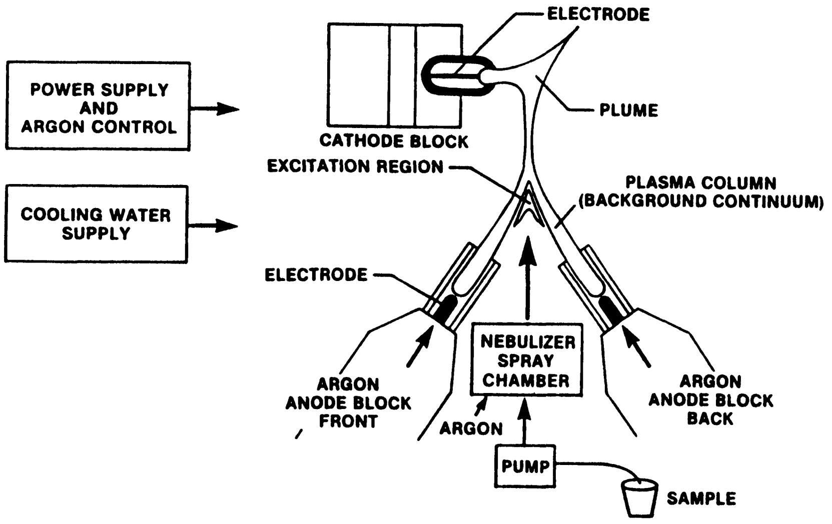

*Figure 2. The SpectraMetrics three-electrode jet.*

The instrument has a Czerny–Turner type monochromator, consisting of a prism and an echelle grating, which projects the spectrum onto the exit slit. For the wavelength range of 200 to 800 nm, reciprocal linear dispersions vary within 0·062 to 0·25 nm/mm. This high resolution virtually eliminates all errors originating from spectral overlap, offering precision and accuracy which is better than that obtained by flame emission methods, and improved detection limits for difficult-to-excite elements [17]. The high resolution also permits the use of fairly wide slit heights and widths (for example 500 × 200 μm), resulting in relatively strong signals in the photomultiplier.

*Configuration of the FIA/DCP-OES system*

In the combined FIA/DCP-OES system, the liquid sample containing the element of interest is injected into a continuous stream of the carrier liquid and is transported to the plasma jet for atomization. On its way to the detector the sample plug is mixed with the carrier liquid and is partially dispersed. The degree of dispersion depends on the distance of injection point from the plasma, on the volume of the sample, on the flow rate and on the inner diameter of the tubing [18]. These parameters have to be optimized for particular samples, solvents and carrier liquids; the viscosities of the latter two also having major roles. In a given procedure experimental conditions have to be kept constant for both samples and standards. The sample concentration can be evaluated against properly made-up standards, which are injected the same way as the samples. Flow injection systems are characterized by short response times; analytical signals are obtained in between 2 s and 5 s which can lead to a high sample throughput.

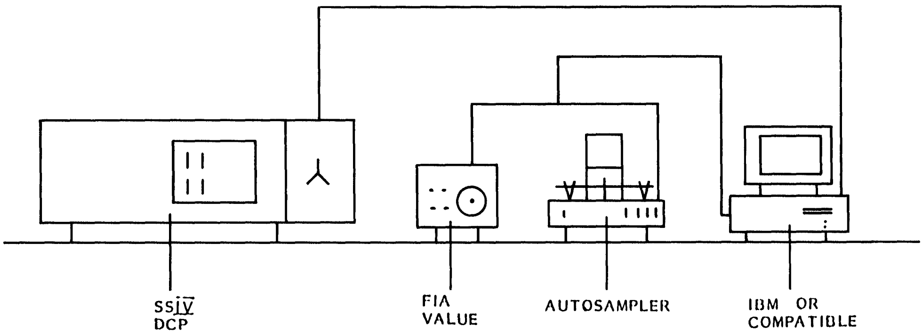

*Figure 3. Schematic diagram of the automated FIA/DCP-OES analyser.*

The schematic diagram of the automated FIA/DCP-OES system is shown in figure 3: it consists of an autosampler, the FIA device, the DCP-OES and a microcomputer. The carrier liquid is transported using a 'Miniplus 3' peristaltic pump, at a rate predetermined to suit the particular analysis of interest. Teflon tubing (internal diameter 0·8 mm) is used where appropriate; however, silicone tubing is applied at the roller heads to accommodate both organic and inorganic solvents. Samples are introduced by the autosampler through a loop. The size of the loop is variable; for most applications a 600 µl loop is sufficient.

A precision-controlled flow injection valve is necessary to keep the dispersion to a minimum and to keep the volume and length of the sample plug as low as possible. A Teflon six-port valve (obtained from PS Analytical, Kemsing, Kent, UK; catalogue number PSA 60.043) was found to be best for the purpose. A schematic flow diagram of the valve configuration for filling and injecting is shown in figure 4. Two ports are used for the loop, one is shown for the inlet and one for the outlet of the carrier stream. The fifth port is used for carrying the plug to the DCP-OES detector, and the sixth port is used as an excess drain. This valve can be controlled manually through an electric switch or, as we recommend, by a microcomputer. The intelligent autosampler (PS Analytical; catalogue number PSA 20.080) is also controlled by the microcomputer.

The FIA valve is connected after the pump approximately 25 cm from the nebulizer to eliminate dispersion as far as possible (this is the shortest distance attainable between the valve and the DCP jet). When attempting a new procedure, a 15 s wash-out period should be allowed between injections; this was found to be adequate to minimize memory effects due to memory-prone elements. However, the time can be shortened to 1–2 s for most applications, once the optimal experimental conditions have been established.

All the experimental parameters can be stored in the microcomputer, and all of the operations can be controlled by it.

*Signal acquisition and the data management system*

This system consists of a microcomputer (ESR class) with RS 232 interface, an enhanced graphics adaptor screen, an Epson 80X printer and a Mettler G15 chart recorder.

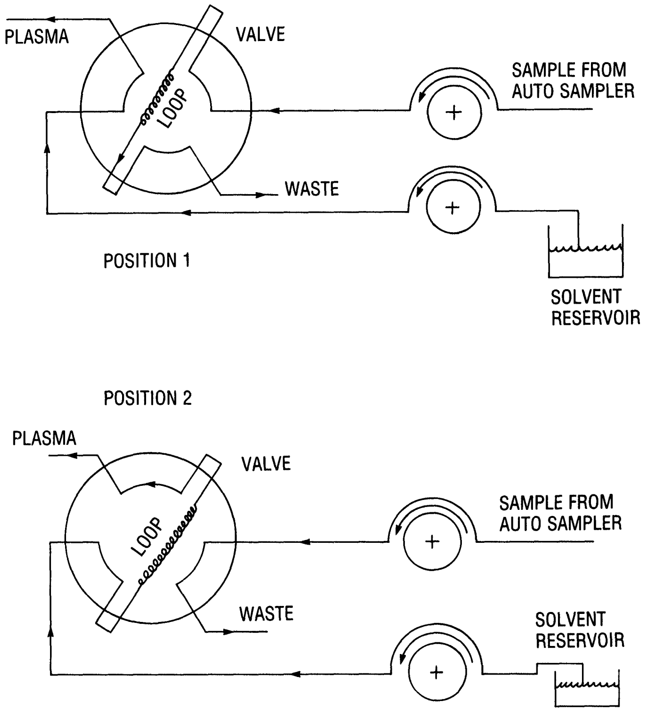

*Figure 4. Schematic flow diagram of a flow injection valve in filling and injecting configurations.*

The 'Touchstone' software (available from PS Analytical; PSA 30.00) data acquisition program is designed to operate with an IBM, or compatible, computer working under MS DOS or a similar operating system. The program is user friendly and follows a step-by-step guide through its facilities. It can collect, store and process data generated as line intensities in the spectrometer. Data are transferred through the serial asynchronous communications interface continuously. When measurements for standards are completed, be it either by standard addition or standard curve, a curve will automatically be displayed on the screen to suit the type of analysis. The program may be interrupted at any time for lodging additional data or information. The data collection system allows statistical analysis to be carried out on a series of similar measurements of different samples, calculating means and standard deviations. A similar option is available for the statistical analysis for repetitive measurements on the same sample.

```

Saved> DCP-TIN.MTH - TIN IN SILICONES. TIN>TIN          ISample Tag   Ref Dc

IMMMMMMMMMMMMMMMMMMMMMMMMMMMMMMMMMMMMMMMMMMMMMMMMMMMMMMMMMMMMMMMMMM9           0

: Library  Method  Calibrate  Options  Analyse  Results  : 29 Aug 89 12:22:06

HMMMMMMMMMMMMMMMMMMMMMMMMMMMMMMMMMMMMMMMMMMMMMMMMMMMMMMMMMMMMMMMMMMMMMMJMMMMMMMMMMMMMMMMMMMMMM

IMMMMMMMMMMMMMMMMMMMMMMMMMMMMMMMMMMMMMMMMMMMMM;

:Method: TIN IN SILICONES.                    :

:Curve Fit by Equal Weight Slope Average:

:Measured by Pk Area                          :

:       0-1V Interface                        :

:  Flow Injection Valve Setup                 :

:DELAY        MEASURE        RESET            :

:20sec        30sec          10sec            :

:Loop:500ul       Fill Rate: 3 ml/min         :

:                                             :

:Instrument: SpectraScan 4                    :

:Wavelength 123.4nm  View:glorious            :

:Nebuliser: Crossflow                         :

:Sample Uptake Rate: ?                        :

:Argon Pressure: 10 lbs/sq in                 :

:Carrier:  1 l/min   Sheath:1.5 l/min         :

:Plasma Sleeve Gas:3.1 l/min                  :

:Nebuliser Input Soln 1: Glacial Acetic       :

:Sample Soln 2: Tin in HCL                    :

```

*Figure 5. Typical template of experimental parameters as displayed by the 'Touchstone' I/O program (showing conditions for the determination of Sn in silicone oils).*

A typical template used in setting up and recording the parameters in method developments is shown in figure 5, listing the experimental and data processing parameters applied in the analysis of tin-based catalysts used in silicone products. The template can be recorded and referred to at any time, and can be easily altered. It contains a general title, indicates the method by which data were processed, followed by the interface voltage which is defined by the intensity of the signal. It shows the delay time applied before activating the valve, to allow thorough cleaning of the carrier line prior to the sample plug injection, followed by the period required for the measurement proper, and a reset time. The remaining items show other important experimental settings. All these values can be entered from the keyboard; they can be altered, and, of course, stored for further use and reference.

A list of the options available in this program are shown on figure 6. This is the print-out of the menu which appears on the computer screen. Any of the listed options may be selected by pressing the initial letter and can be abandoned by using an escape key. The program allows storage of results under a file name or 'Tag' for easy retrieval and editing. Print-out is also incorporated into the program. When the test is completed, it is possible to go into an alternative file without returning to the main menu. The print facility is elaborate and can print-out data, diagrams and formal reports.

### Results, discussion and conclusions

The technique developed allows a rapid and routine determination, both at trace and higher concentration levels, either in aqueous or non-aqueous solvents, of approximately 70 elements in the periodic table. It is fully automatic and all the operations (including sampling and clean-out of the plasma jet) are controlled by a microcomputer. The facility of controlling the time allowed for clean-out of the plasma jet was especially useful when

```

IMMMMMMMMMMMMMMMMMMMMMMMMMMMMMMMMMMMMMMMMMMMMMMMMMMMMMMMMMMMMMMM9                 0   0:

Library  Method  Calibrate  Options  Analyse  Results  : 29 Aug 89 12:18:40  :

HMMMMMMMMMMMMMKMMMMMMMMMMMMMMMMMMMMMMMMMMMMMMMMMMMMMMMMMMMMMMMMMJMMMMMMMMMMMMMMMMMMMMMMM<

:Select          :Reslope           :Single          :Display

:List            :Curve Plot        :Batch           :Print

:Add             :Amend Curve       :Program         :Backup

:Replace         :New Curve         :Change Tag      :Amend

:Erase           :Std Addition      :Make Tag        :Weight/Dil

:Disc Read                          :Runs            :Store

                                    :Timer Set       :Retrieve

        :Settings          :Autosampler              :Full Report

        :Notebook          :Printer                  :Lotus file

        :Interface         :Beep                     :Erase

        :Transfer          :Timer                    :Clear Screen

        :Filter            :Key Input                :Quit

                           :Save Settings

```

*Figure 6. List of facilities available in the 'Touchstone' I/O programme.*

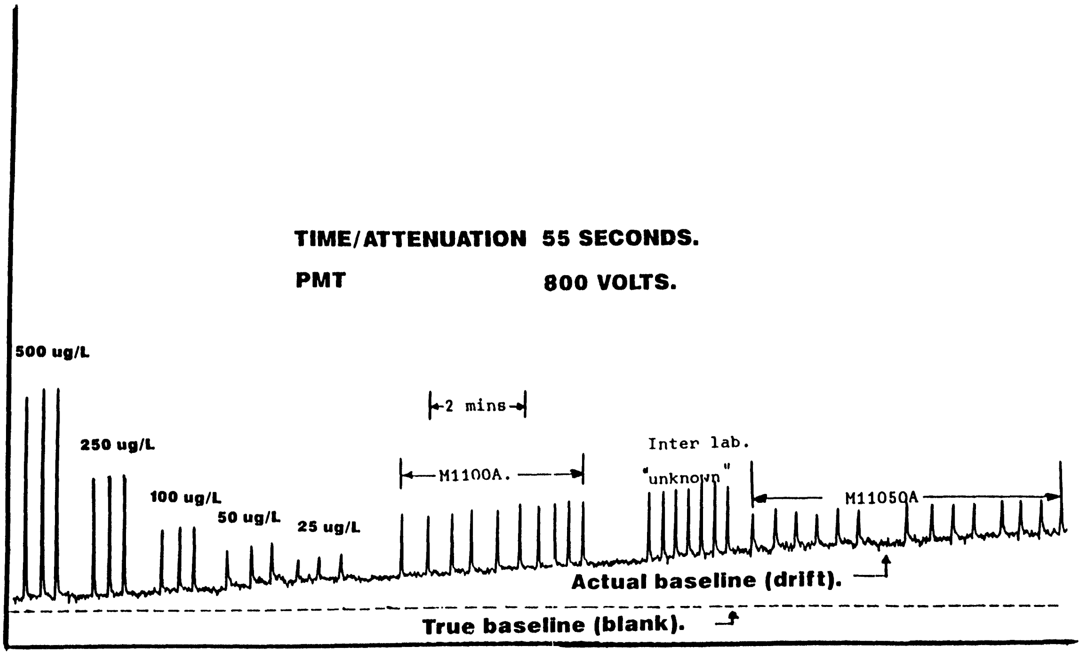

*Figure 7. Reproducibility of signals with a shifting base-line.*

determining elements which are prone to 'memory effects' from previous samples or standards. Thus, with the determination of difficult elements, including tungsten, boron and molybdenum, this technique produced substantially more precise and accurate results than traditional batch operation. The DCP-OES parameters can be set to various levels to accommodate widest concentration ranges. Thus, a high attenuation setting (900 V) can be used for trace analysis, while a lower value (500 V) will suit samples which contain only a small fraction of the analyte being investigated.

The flow injection method was extremely valuable in correcting for base-line drifts (which originate from uncontrollable thermal and electronic changes during the course of operations). This drift can cause high errors, especially at trace levels, when using the conventional continuous nebulization technique. The elaborate and time-consuming correction procedures, requiring the intermittent spraying of high and low standards are not necessary with the FIA method; when the base-line is defined by the emission obtained from the carrier liquid, and is reproduced between each sample injection. Figure 7 illustrates the point well. Note that the peak heights, when measured from the base-line (as done in the FIA technique), are very reproducible; if, however, these peaks were measured from the initial blank value (called on the figure the 'true' base-line) substantial errors would occur.

Automation is especially advantageous if large numbers of samples must be analysed on a routine basis. The flow injection method requires low sample volumes, but even the recommended 600 μl loop size can be reduced to approximately 100 μl without substantial losses in sensitivity, accuracy or precision; in certain applications involving ICP-s as little as 20 μl samples have been suggested [19]. There is little doubt that sample introduction using a flow injection valve and propelled by a peristaltic pump or another solvent delivery pump is superior to other micro sampling techniques [20]. Flow injection analysis also offers a number of other advantages, such as the potential for providing a complete analytical calibration curve from a single standard solution [21] – the exponential decay of the signal effectively provides a calibration with an infinite number of points. Most of the equipment mentioned here can be used in combination with other instrumental analytical techniques (ICP-OES, atomic absorption spectrometry, UV-visible molecular absorption spectrometry, spectrofluorimetry and voltammetry). Compared with the cost of the instruments and the operators' time, the setting up and operation of the technique is relatively inexpensive.

In general, one can say that flow injection methods are quicker, more precise and use less reagents than other techniques; in addition, they are very applicable where only a limited amount of sample is available. The main advantages of the combination of FIA with DCP-OES are the increase in precision in sample handling, fewer physical interferences, a higher throughput of samples and versatility towards physical and chemical properties of reagents. Some disadvantages have to be mentioned as well; for example the loss in sensitivity compared with continuous nebulization and the fact that only one element can be analysed at a time: this latter problem is being addressed in future work.

The work originally developed in the research and development area has been readily transferred to a quality assurance environment. This shows that the modular flexibility of the 'Touchstone' software in association with various modules allows the user to configure tailor-made solutions to real-world analytical problems. In this respect, the ruggedness of the DC Plasma spectrometer greatly extends the usefulness of the modules and drastically reduces sample preparation procedures which are time-consuming and prone to errors. These several points ensure that the precision of the analytical procedures designed are adequate for the task in hand.

### Acknowledgement

'Touchstone' is a trademark of Spinoff Technical Systems and the co-operation of the proprietor, Mr J. R. Hall, is gratefully acknowledged.

### References

1. Jacintho, A. O., Zagatto, E. A. G., Bergamin Filho, H., Krug, F. J., Reis, B. F., Bruns, R. E. and Kowalski, B. R., *Analytica Chemica Acta*, **130** (1981), 243.
2. Zagatto, E. A. G., Jacintho, A. O., Krug, F. J., Reis, B. F., Bruns, R. E. and Araujo, M. C. U., *Analytica Chimica Acta*, **145** (1983), 169.
3. Israel, I. and Barnes, R. M., *Analytical Chemistry*, **56** (1984), 1188.
4. McLeod, C. W., Cook, I. G., Worsfold, P. J., Davies, J. E. and Queay, J., *Spectrochimica Acta*, **40B** (1985), 57.
5. LaFreniere, K. E., Rice, G. W. and Fassel, V. A., *Spectrochimica Acta*, **40B** (1985), 1495.
6. Elgersma, J. W., Maessen, F. J. M. J. and Niessen, W. M. A., *Spectrochimica Acta*, **41B** (1986), 1217.
7. Lawrence, K. E., Rice, G. W. and Fassel, V. A., *Analytical Chemistry*, **56** (1984), 289.
8. Hartenstein, S. D., Ruzicka, J. and Christian, G. D., *Analytical Chemistry*, **57** (1985), 21.
9. Thompson, J. J. and Houk, R. S., *Analytical Chemistry*, **58** (1986), 2541.
10. Gustavsson, A., *Spectrochimica Acta*, **42B** (1987), 111.
11. Gallego, M., Luque de Castro, M. D. and Valcarel, M., *At. Spectrosc.*, **6** (1985), 16.
12. Fang, Z., *Fenxi Huaxue*, **14** (1986), 549.
13. McLeod, C. W., *Journal of Analytical Atomic Spectrometry*, **2** (1987), 549.
14. Brennan, M. C. and Svehla, G., *Z. anal. Chem.*, **335** (1989), 893.
15. Ruzicka, J. and Hansen, E. H., *Flow Injection Analysis* (2nd edn., Wiley – Interscience, Chichester, 1988), 20 ff.
16. Skoog, D. A., West, D. M. and Holler, J. M., *Fundamentals of Analytical Chemistry* (5th edn., Saunders College Publishing Int. Ed.), 574.
17. *Instruction and Training Manual Handbook* (SpectraMetrics Inc., Andover, Massachusetts, USA).
18. Ruzicka, J. and Hansen, E. H., *Analytica Chimica Acta*, **99** (1973), 37.
19. Faske, A. J., Snable, K. R., Boorn, A. W. and Browner, R. F., *Applied Spectroscopy*, **39** (1985), 542.
20. Tyson, J. F., *Analyst*, **110** (1985), 419.
21. Greenfield, S., *Ind. Res. Dev.*, **23** (1981), 140.

